# Review of the COVID-19 Risk in Multiple Sclerosis

**DOI:** 10.33696/immunology.3.080

**Published:** 2021

**Authors:** Farhan Chaudhry, Cristina Jageka, Phillip D. Levy, Mirela Cerghet, Robert P Lisak

**Affiliations:** 1 Department of Emergency Medicine and Integrative Biosciences, Wayne State University School of Medicine, Detroit, MI, USA; 2 Department of Neurology, Henry Ford Health System, Detroit, MI, USA; 3 Department of Neurology, Wayne State University School of Medicine, Detroit, MI, USA; 4 Department of Neurology, Wayne State University School of Medicine and the Detroit Medical Center, Detroit, MI, USA

## Abstract

The ongoing pandemic of the novel coronavirus of 2019 (COVID-19) has resulted in over 1 million deaths, primarily affecting older patients with chronic ailments. Multiple sclerosis (MS) patients have been deemed particularly vulnerable given their high rates of disability and increased susceptibility to infections. There have also been concerns regarding disease-modifying therapy (DMT) during the pandemic as many DMTs may increase the risk of infection due to some of their immunosuppressive properties. Furthermore, due to MS-related chronic inflammatory damage within the central nervous system, there have been concerns for worsening neurological injury by COVID-19. This has resulted in an alarmingly high level of anxiety and stress among the MS community leading to a lack of compliance with medications and routine check-ups, and even failure to obtain treatment for relapse. However, there is currently substantial evidence that MS and most DMT usage is not associated with increased COVID-19 severity. MS patients who suffer worse outcomes were more likely to be older and suffer from significant disabilities and comorbid conditions, which would also be expected from those in the general population. Likewise, there is little if any evidence demonstrating an increased susceptibility of MS patients to COVID-19-related neurological complications. Therefore, we aim to summarize the most recent findings related to COVID-19 and MS demonstrating that MS and most DMTs do not appear as risk factors for severe COVID-19.

## Introduction

Severe respiratory syndrome coronavirus-2 (SARS-CoV-2) is the virus responsible for the novel coronavirus disease of 2019 (COVID-19) and has resulted in the death of over one million people around the world [1]. COVID-19’s presentation is highly heterogeneous as cases range from asymptomatic to rapidly progressive resulting in low survival rates [2,3]. Specifically, patients that are older, have multiple comorbidities (i.e hypertension, lung disease, diabetes, obesity), and reside in nursing homes are more likely to succumb to COVID-19 [1–4]. Black/African Americans have a higher risk of COVID-19 severity [5]. This is most likely multifactorial, stemming from healthcare disadvantages associated with socioeconomic disadvantages suffered among this population. Therefore, we have become particularly concerned during this pandemic for our patients with chronic debilitating diseases who are more likely to possess these risk factors.

Multiple sclerosis (MS) is a common chronic immune-mediated demyelinating disease of the central nervous system(CNS) [6]. MS immunopathogenesis involves CNS inflammation, blood-brain-barrier (BBB) disruption, and autoreactive lymphocytes thereby requiring disease-modifying therapies (DMT) of immunomodulation, immunosuppression, cell depletion and/or alteration of inflammatory cell trafficking [6]. MS is categorized into several phenotypes including primary progressive MS (PPMS), relapsing-remitting MS (RRMS), and secondary progressive MS (SPMS) [7]. PPMS is characterized by a continuously steady progression and a higher rate of disability when compared to that seen in RRMS. SPMS patients tend to be older in age than RRMS patients since it evolves from RRMS over the course of 10–20 years and is characterized by the gradual worsening of the disease. Furthermore, due to the chronic and disabling nature of MS, many patients will eventually reside in long-term care facilities [8].

Consequently, the combination of pathogenesis, treatment and natural history most likely explains the four-fold greater risk for contracting serious infections in MS patients when compared to that seen in the general population [9]. We would thus reasonably expect that MS patients are at higher risk of contracting COVID-19 and have worse outcomes [10]. Additionally, due to the compromise of the CNS by MS and the growing evidence of COVID-19 neurological manifestations, there are concerns for higher rates of neurological complications in MS patients [10,11].

**Figure 1:**
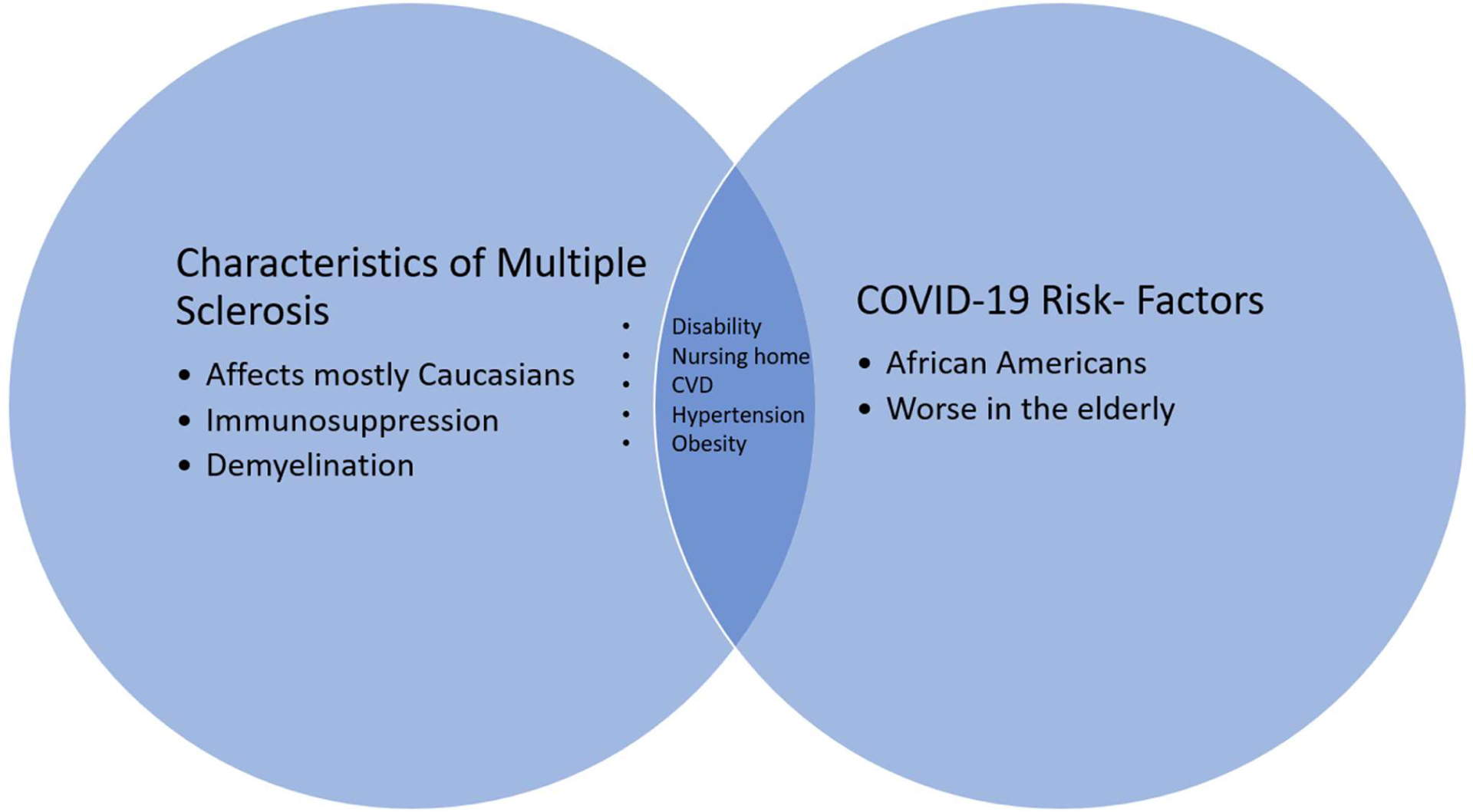
Venn Diagram between Characteristics of Multiple Sclerosis and Risk Factors for COVID-19. The Venn diagram shows known overlaps between characteristics of MS and known risk factors for COVID-19.

Cardiovascular disease and hypertension are significantly associated with COVID-19 risk [12,13]. Patients with MS are at a significantly increased risk for these risk-factors when compared to the general population [14,15]. This is most likely due to higher rates of disability resulting in less healthy life-styles (i.e smoking, obesity, reduced physical activity) [16]. These higher rates of comorbidities could also contribute to worse COVID-19 outcomes in MS patients (Figure 1). Early studies were contradicting as survey reports from China, found no increased risk of COVID-19 in MS patients [10]. Conversely, studies from Spain and France found that MS patients are at a higher risk for poor COVID-19 outcomes when compared to the seen from the general population, but not necessarily at a higher risk to contract COVID-19 [17,18].

Recently, though, evidence from a larger cohort in the United States, Canada, and the United Kingdom actually showed that the risk of severe outcomes from COVID-19 was similar to that seen from the general population [19]. Regardless, all patients with more severe COVID-19 cases were older, and had higher levels of disability [17,18,20]. Additionally, the Black/African American race was also associated with worse COVID-19 outcomes [19]. As mentioned before, these risk factors have been found in the general population as well. Patients with progressive forms of MS were also more likely to succumb to severe infection, but this has yet to be found statistically significant in further larger studies [19,20]. This sub-group, due to the nature of the disease, tend to have worse disabilities and have poorer health, which most likely contributes to COVID-19 severity [21].

**Figure 2:**
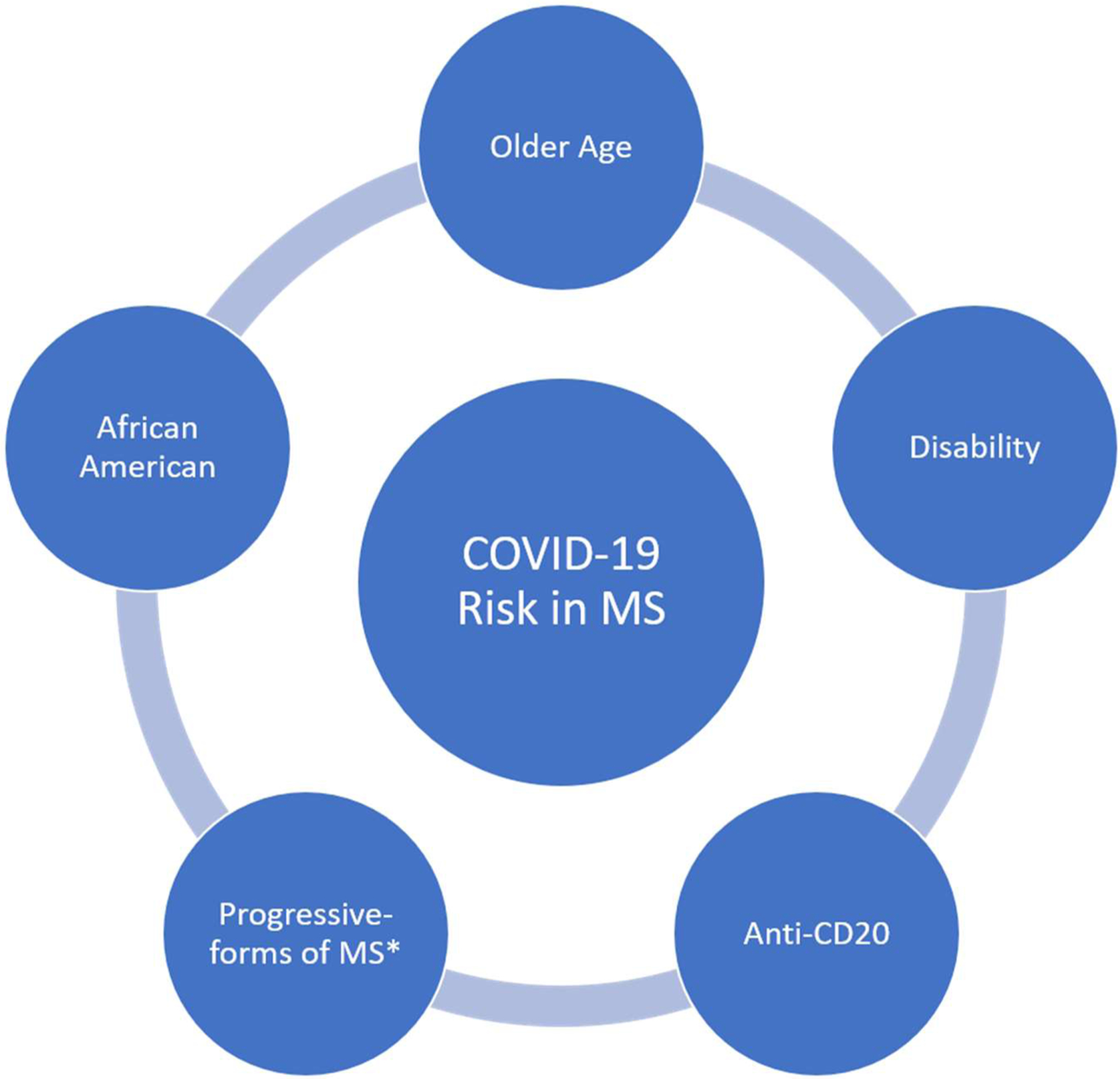
Risk-factors Associated with COVID-19 Risk in MS. Figure shows risk-factors associated with worse COVID-19 outcomes in MS patients. *= Progressive forms of MS have yet to be found to be statistically significant.

Contrary to what was originally thought, DMTs and higher levels of immunosuppression were not independent risk factors for worse COVID-19 outcomes in MS, with the possible exception of anti-CD20 therapy [17,19,20]. Likewise, in other immune-mediated diseases requiring chronic immunosuppression, immunosuppressive therapy has failed to significantly increase the severity of COVID-19 [22–24]. Furthermore, MS patients had symptoms similar to that experienced in the general population, without a higher rate of neurological complications. The concern for MS patients during the COVID-19 pandemic is still warranted with respect to those who are older, have more comorbidities and have significant disability, but we should bring into question MS and DMTs as severe COVID-19 risk factors (Figure 2). In this review, we aim to summarize clinical and basic science data surrounding COVID-19 and MS.

## COVID-19 Pathogenesis

### Virology

SARS-CoV-2 is an RNA-virus of the same genus as the severe acute respiratory syndrome coronavirus of 2002–2003 (SARS-CoV) and targets angiotensin-converting enzyme-2 (ACE2) with its spike-glycoprotein [25]. SARS-CoV spike-glycoprotein utilizes the serine protease TMPRSS2 for priming to enter ACE2+ cells [26]. Targeting of ACE2 with spike-glycoprotein results in significant acute lung injury via an excessive inflammatory response mediated by an imbalance of Th17/Treg cells and an overproduction of proinflammatory cytokines [27–29].

### Possible cytokine storm

This cytokine-storm-like response in COVID-19 was supported by early reports demonstrating COVID-19 severity was associated with increased plasma levels of cytokines such as IL-6 [30–32]. Postmortem studies of COVID-19-infected lungs also found significant signs of an inflammatory response dominated by lymphocytes resulting in severe lung damage [33,34]. Furthermore, preliminary results from clinical trials provide evidence that glucocorticoids can significantly improve COVID-19 outcomes in moderately and severely affected individuals, possibly by reducing this inflammatory response [35,36].

The cytokine-storm hypothesis stemming from COVID-19, however, has recently been brought into question. It was shown in a small cohort of patients that cytokine levels in severe COVID-19 cases were in fact lower than that seen from patients with bacterial sepsis and similar to that seen in other critically ill patients [37,38]. Furthermore, targeting IL-6 with tocilizumab was found not to be effective in preventing severe COVID-19 disease [39]. Nonetheless, IL-1 inhibition with Anakinra was associated with reduced rates of severe COVID-19, but clinical trials are still ongoing (NCT04603742) [40]. Cytokine storms are multifaceted with many different mediators, both cytokine and non-cytokine in nature. Indeed, the failure of an inhibitor of one of many proinflammatory cytokines does not negate the idea that a mixture of proinflammatory cytokines produces deleterious clinical outcomes. Therefore, even though there is evidence for a cytokine storm-like response in COVID-19, it may not adhere to the same pathogenesis typically seen in a cytokine storm.

## DMT and Infection Risk

### Types of MS therapies

There are many different treatment options with different mechanisms of action that are used for MS [41]. Relapse treatments include glucocorticoids and occasionally plasmapheresis in corticosteroid failures; however, these are typically reserved for acute relapses of MS, thus are administered only for a short term. DMTs, on the other hand, are administered chronically to help prevent relapse thereby slowing down MS progression. Detailed reviews of DMTs have previously been written [41,42]. DMTs are known to reduce relapses and slow the progression of disabilities by adjusting and/or suppressing the immune system, thus reducing the formation of new CNS lesions.

Classification of DMTs, albeit imperfect and sometimes not fully understood as many DMTs have many different mechanisms of action, can be divided into: 1) immunomodulation: interferon-beta-1 (IFN-b1), glatiramer acetate (GA), and fumarates (i.e dimethyl fumarate), 2) cell trafficking alterations molecules like S1P receptor modulators (i.e fingolimod), and natalizumab, an anti-α4-integrin antibody, 3) cell depletion (anti-CD20 antibodies [i.e ocrelizumab, rituximab, ofatumumab], cladribine, and anti-CD52 antibodies [i.e. alemtuzumab]), and 4) systemic immunosuppression (i.e teriflunomide) [41,42].

### DMTs and overall infection risk

Among DMTs, GA and IFN-b1 were associated with a 50% increase risk of overall serious infections (defined as an infection resulting in hospitalization) in MS patients in comparison to that seen from age-/sex-matched non-MS patients [43]. However, this study most likely showed the effect of the higher risk of infection seen in MS patients as increases in overall infection was not prominent in clinical trials of these agents studied only in MS patients [44–46]. Furthermore, GA and IFN-b1 usage were not associated with higher rates of physician-reported infection-related claims for MS patients when compared to that seen from no DMT usage [47].

In head-to-head comparison studies, the anti-CD20 antibody rituximab had a significantly higher rate of overall severe infection in comparison to that seen from GA and IFN-b1 in MS patients [43]. There was also a slightly higher rate of overall severe infection with rituximab when compared to that seen in natalizumab and fingolimod treated MS patients, but this was not statistically significant [43]. Similarly, MS patients treated with the newer anti-CD20 antibody, ocrelizumab, were twice as likely to suffer from an upper respiratory infection compared to placebo-treated patients [48]. Fingolimod and natalizumab also had higher rates of severe infection when compared to IFN-b1 and GA, but not statistically significant [43]. Natalizumab was associated, with a 59% higher relative risk of infection-related physician claims compared to that seen from MS patients not on DMT [47]. Herpes zoster infections were more frequent in the cladribine-treated arm compared to that seen in the placebo-arm in an MS clinical trial [49]. Herpes simplex infections were more frequent in MS patients treated with the anti-CD52 antibody, alemtuzumab, in comparison to those treated with IFN-b1 [50].

### DMT exposure and COVID-19 risk

Given that immunosuppression by some DMTs further increases the risk of infection in MS patients, it was reasonable to assume that this would pertain to COVID-19 as well. Rituximab and ocrelizumab were especially concerning due to their well-established infection-risk profile [43]. By targeting CD-20, B-cells are largely eliminated in the peripheral immune system, reducing B-cell cytokines and availability of B cells to act as antigen-presenting cells. Reduction of B-cell development and differentiation to plasma cells also occurs, but the early therapeutic effects do not seem to involve the reduction of serum and cerebrospinal fluid (CSF) IgG [51]. Importantly, anti-CD20 antibodies reduce type-II pneumocyte response to infections and prevents CD4+ T-cell priming, thereby attenuating the clearance of viral infections from the respiratory tract [51,52].

Recent evidence, however, fails to show that most DMTs are independent risk factors for COVID-19 incidence and severity [17,19,20,53]. In fact, some hypothesized that DMTs may in fact be protective against COVID-19 by attenuating the cytokine-storm-like response [54,55]. Additionally, certain DMTs (i.e GA, fumaric acid, fingolimod) are associated with an increased expression of circulating natural killer cells possibly allowing for a better defense against COVID-19 [56]. These theories, though, are still purely speculative and subgroup analyses of different DMT therapies, albeit small in sample size, have yet to show protection from COVID-19 [17,20,53].

Analysis of a larger dataset of COVID-19 MS patients did, however, reveal that anti-CD20 treatment may portend worse COVID-19 outcomes [19]. Rituximab-usage resulted in significantly higher hospital admissions (aPRs=1.58, P<0.05), ICU admissions (aPR=4.12, P<0.05), and mechanical ventilation (aPR=7.27, P<0.05) when compared to that seen with dimethyl fumarate-usage among MS patients [19]. Ocrelizumab was only associated with more ICU admissions (aPR=3.53, P<0.05) when compared to dimethyl fumarate, but not associated with more total hospital admissions and mechanical ventilations [19]. Overall, pooled-frequencies of hospitalizations, ICU admissions and ventilations were significantly higher for MS patients treated with anti-CD20 antibodies when compared to those treated with other DMTs (aPR=1.49, 2.55, and 3.05 respectively, all P<0.05). The relatively higher association of rituximab with poorer COVID-19 outcomes in comparison to that seen with ocrelizumab, admittedly not a head-to-head study, could be because rituximab has a significantly higher affinity, resulting in more deleterious effects on B-cell numbers [57].

### Anti-CD20 therapies’ possible effect on the COVID-19 vaccine

There are many COVID-19 vaccines under development, however, Pfizer’s BNT162b vaccine was the first one approved for administration in the U.S and has been shown to be highly effective [58]. Since anti-CD20 antibodies deplete B-cells, there is some concern regarding their potential to attenuate humoral immune responsiveness to COVID-19 vaccines [59]. Both rituximab and ocrelizumab have been shown to blunt influenza vaccine seroconversion [60,61]. BNT162b, conversely though, is a unique mRNA-based vaccine resulting in a robust CD8+ T-cell response in addition to a humoral response, thereby possibly circumventing the attenuating effects from anti-CD20 antibodies [62]. Consequently, we would expect that BNT162b would still be highly efficacious despite anti-CD20 treatment. Even if anti-CD20 antibodies blunt BNT162b response, the risks associated with BNT162b are minimal, thus the benefit of BNT162b would most likely still far-exceed the risks [58]. Moderna’s mRNA-1273 vaccine was also approved for use in the US and has been confirmed to be effective against COVID-19. It is also an mRNA virus and would work in a similar fashion as the BNT162b vaccine, thus, having a similar risk-benefit ratio for MS patients [63].

## Neurological Implications of COVID-19 in MS

### COVID-19-associated neuropathogenesis

SARS viruses have been known to invade the brain [64]. ACE2 is expressed along the cerebrovasculature comprising part of the BBB, facilitating SARS transport via spike-protein targeting [65]. *In vitro* models show that SARS-CoV-2 spike protein, once bound to ACE-2, can disrupt the BBB by increasing the pro-inflammatory response by endothelial cells [66]. Specifically, endothelial cells when exposed to the spike-protein increased expression of ICAM-1 and VCAM-1 allowing increased leukocyte adhesion to the endothelium, and also increased expression of IL-1b, IL-6 and CCL5. It does not appear that the spike-protein is cytotoxic to the endothelial cells itself, but it does increase BBB permeability most likely due to increased expression of matrix metallo-proteinases (MMPs), thereby possibly permitting invasion by inflammatory cells.

There have been an alarming number of neurological manifestations of COVID-19 ranging from mild, such as headaches and myalgia, to severe, such as stroke and encephalopathy [67,68]. Whether direct infection or secondary consequences of COVID-19 such as its known hyper-coagulopathic effect is the reason for some of its neurologic-implications remains to be explored [69]. Nonetheless, the potential implications of COVID-19 on the CNS could theoretically exacerbate pre-existing neurologic injury stemming from MS.

## COVID-19 in the MS Brain

BBB breakdown is a part of the pathogenesis of MS. The breakdown of the BBB in MS is mediated by inflammatory injury and the release of MMPs [70]. The breakdown of the BBB results in immune cell infiltration promoting demyelination. COVID-19 results in a systemic inflammatory response and induces vascular damage, thereby potentially exacerbating BBB breakdown and worsening MS progression [71]. Case reports/case-series have detected COVID-19 in MS patients with possible relapse, but only one case detected SARS-CoV-2 within the CSF [72–74]. Furthermore, disability only increased mildly (2–3 points on the Extended Disability Status Score), there were no MRI-confirmed findings of relapse, and all patients made a full neurological recovery. Therefore, these were likely not a true relapse, but what has been termed as pseudo-relapse. Pseudo-relapse is the transient worsening of MS–symptoms due to something other than true-demyelinating pathogenesis [75]. They are triggered by other processes such as fever, heat exposure or infection/inflammation [76].

It is possible that it is too early to tell, and studies have been too small to detect a noticeable increase in neurological symptoms. However, this means that the chance of neurological complications in MS patients is low, and probably like that seen from the general population.

### Viral molecular mimicry in MS

Certain viruses have been implicated in MS. The risk of MS has been shown to significantly increase in those with certain genetic susceptibilities who have had EBV infection [77]. Therefore, it is hypothesized that EBV infection in certain genetically susceptible patients can cause a molecular mimicry autoimmune response leading to MS, though this has yet to be substantiated [78].

The role of molecular mimicry by COVID-19 leading to neurological injury has been considered because of the reported association between COVID-19 and Guillain-Barré syndrome(GBS), an immune-mediated demyelinating disease of the peripheral nervous system [79,80]. Due to the significant relationship between GBS and infections, molecular mimicry has been strongly implicated in the pathogenesis of GBS, but most notably with bacterial infections of *Campylobacter jejuni*. However, the potential relationship between COVID-19 and GBS is only based on small case-series/studies and require larger epidemiological analysis [81]. Nonetheless, the potential molecular mimicry from COVID-19 has gained tremendous attention [81–83]. Recently, a computational analysis found that 20 human peptides were uniquely mimicked by SARS-CoV-2 [84]. Four of these peptides were mapped onto different human leukocyte antigen (HLA) phenotypes. The most pertinent neurological protein was annexin A7 (ANXA7, also called synexin), which was found to be expressed along with endothelial cells across many different organs including the brain and found to be expressed on oligodendrocytes. Annexins are a family of calcium-binding proteins that are involved in a diverse array of cellular mechanisms vesicle transport to apoptosis [85,86]. ANXA7, specifically, plays a role in vesicle exocytosis, the release of glutamate, and NMDA trafficking [87]. In the presence of intracerebral hemorrhage (ICH), ANXA7 exacerbates the release of glutamate and the trafficking of NMDA receptors, thereby potentially worsening glutamate excitotoxicity and secondary brain injury. This could support a theory that there can be molecular mimicry of SARS-CoV-2 targeting ANXA7-expressing cells in the brain, resulting in neurological injury [88].

The spike protein of SARS-CoV binds not only to ACE2, but also to sialic acid-containing glycoproteins and gangliosides on the cellular membrane (i.e GM1) [81,89]. It has been speculated that there could be cross-reactivity between epitopes at the spike-ganglioside interface and glycolipids along peripheral nerves [81]. This mechanism of molecular mimicry has been shown as a trigger for GBS following *Campylobacter jejuni* infection [90]. Even though the presence of mimicked peptides does not necessitate an inflammatory response, the role of molecular mimicry in demyelination/neurologic injury stemming from SARS-CoV-2 warrants further research.

## Conclusion

The ongoing COVID-19 pandemic has created a lot of anxiety among MS patients, their families and physicians who care for patients with MS. In comparison to the general population, MS patients have had higher rates of depression, worse sleep quality, and fatigue during the pandemic [91]. Additionally, many MS patients are concerned about their DMT-regimens during the pandemic and some have even stopped it [92].

MS patients were believed to represent a particularly vulnerable group to COVID-19; however, mounting evidence allows us to reassess their presumed vulnerability. MS patients that are at increased risk tend to be older, have more disability, and have comorbidities such as obesity, hypertension, cardiovascular disease, diabetes and others that come with increasing age, which are similar to that seen in the general population. Currently, these risk factors appear to represent the consequences of MS and patients’ associated comorbidities rather than MS itself. Except for some anti-CD20 therapies, the use of DMT does not seem to be a risk factor for COVID-19 in MS. Therefore, MS and its treatments are most likely not strong risk factors for COVID-19, contrary to what was once thought. Instead, we should start tailoring our risk-models to look at the characteristics of our MS patients individually.
